# Diagnostic reference levels in Africa: a protocol for systematic review and meta-analysis

**DOI:** 10.4314/ahs.v25i1.15

**Published:** 2025-03

**Authors:** Ademola Joseph Adekanmi, Bidemi I Akinlade, Owowunmi Alex Adedoyin

**Affiliations:** 1 University of Ibadan College of Medicine, Radiology; 2 University of Ibadan College of Medicine, Radiation; 3 University College Hospital Ibadan Department of Radiology Research office

**Keywords:** Diagnostic reference levels, Medical exposure, Radiation Dose, X-ray medical examination, Radiation dose, Radiography, Radiology, Biological effects

## Abstract

**Background:**

Despite its wide acceptance and recommendation as an essential tool for radiation exposure optimization and the increasing influx of high-technology imaging facilities, there is still a paucity of available data on the Diagnostic reference level (DRL) of ionizing radiation radiological examinations and its use in many African countries.

**Objectives:**

This review aims to evaluate published work on DRLs in Africa and to establish the status of DRL in common radiological examinations in Africa.

**Methods:**

The electronic databases, namely; Embase, PubMed, Scopus, Google Scholar, CINAHL, AJOL, and Web of Science, will be searched using a developed search strategy to include only published articles and survey studies retrieved in English from 1996 to December 2022. Information to be extracted will include the World Bank income level, World Bank geographical region, country of origin, research sponsorship, year of publication, age group of the patients, imaging modality, local/regional/national DRL, type of examination, study design, type of DRL quantity, and whether DRLs were established as the 75th percentile or mean of the median or mean of DRL quantity measured. Descriptive statistics will be formulated and a narrative synthesis of the information from selected studies measured. If required, we will include a subgroup analysis based on the income level of the countries, geographical regions, and year of publication.

**Results:**

This study will provide information on the status of diagnostic reference levels of common radiological examinations in Africa.

**Conclusion:**

The result of this study will be useful in healthcare policymaking and by the end-users of medical radiation facilities, thereby ensuring the optimization of radiation exposure in patients undergoing medical ionizing-radiation imaging.

## Introduction

Diagnostic medical imaging is an essential element of any healthcare system. It plays an important role in the diagnosis, treatment, and prognosis of numerous diseases in almost all medical fields^1^. Over the years medical imaging has emerged as an important component of universal health coverage (UHC). Although in Africa, the population's access to medical imaging using ionizing radiation and the distribution of radiology equipment is still inadequate, there has been an increase in the utilization of medical imaging using ionizing radiation in recent years, improving patient management and treatment outcomes^2^,^3^.

However, if appropriate dose-reduction strategies are not undertaken, the increased use of medical imaging for diagnostic and interventional radiology may significantly increase the ionizing radiation exposure level in the population^4^. To ensure safety in medical imaging, the principles of justification and optimization are commonly employed through the use of clinical imaging guidelines and dosimetry of patients undergoing routine diagnostic examinations to assess the level of exposure.

Diagnostic reference level (DRL), an important component of medical imaging optimization was introduced by the International Commission on Radiological Protection (ICRP) to ensure safety and promote quality assurance in medical imaging of various procedures using ionizing radiation^5^,^6^. The diagnostic reference level is defined by ICRP as a form of investigation level, applied as an instrument to aid optimization of protection in the medical exposure of patients for diagnostic and interventional procedures^5^. Diagnostic reference level is a specified radiation dose for a given imaging study that is not expected to be exceeded, otherwise, an investigation into the image acquisition technique or equipment performance has to be undertaken.

The objective of the DRL process in radiology is to evaluate whether the amount of ionizing radiation applied for a medical imaging procedure under defined conditions, is too high or too low^5^,^7^. Generally, the DRL process assists in the optimization of procedure-specific radiation dose levels, determination of excessive radiation, and comparisons between different equipment and protocol guidelines, as well as in the provision of devices for adjusting patients' ionizing radiation exposure^7^–^9^. Although, the DRL process alone may be inadequate for optimization of protection in radiology as the foremost priority for any clinical imaging examination is to achieve quality diagnostic information adequate for the clinical purpose, the DRL has proven to be an effective device that assists in the optimization of protection in the medical exposure of patients for diagnostic and interventional procedures^5^,^8^,^10^.

Diagnostic reference level (DRL) has become a fundamental aspect in the optimization of medical imaging. The establishment of DRL values and implementation of the DRL process has proven to be an effective mechanism for quality control and dose reduction^5^,^6^. The ICRP publications 60, 73, 105, and 135 and ICRP Supporting Guidance 2 reports provided guidance for the establishment and implementation of DRL values for specific imaging modalities, advice on the periodic revision of DRL values, and recommendations on DRL quantities for use with specific imaging modalities. However, this has not been comprehensively addressed in Africa. Hence, the need for a comprehensive assessment of the current status of DRLs in Africa and to provide a view into the peculiarities associated with DRLs usage. Therefore, this study is meant to establish the current situation/status of DLR in imaging studies looking at available published data on DRLs in Africa. The findings from this review will assess the level of implementation of the DRL process in Africa. Furthermore, it will identify gaps in knowledge and could inform, and encourage future research in this area and also aid policy formulation.

### Objective

This study aims to review available data and the use of Diagnostic reference levels in Diagnostic medical examinations (Radiography, Computed Tomography, Fluoroscopy and Mammography) in Africa.

### Specific objectives of this study include

1. To ascertain available data on Diagnostic dose levels in Radiography, Computed Tomography, Fluoroscopy and Mammography examinations in African countries.
2. To determine the pattern of documentation of DRLs in Radiography, Computed Tomography, Fluoroscopy and mammography examinations in Africa.
3. To identify factors associated with/affecting the usage of various DRLs in Radiography, Computed Tomography, Fluoroscopy and mammography in Africa. These include lack of DRLs standard operating procedure in hospitals/imaging centres, limited human resources, poor prioritization of imaging facilities, partial integration/non-integration of medical imaging into health care services, poor or inadequate awareness of radiation safety, inadequate radiation safety culture, poor equipment state, lack of/insufficient facilities and opportunities for education and training, as well as low per capita healthcare budget.

## Methods

### Study Registration

This protocol of systematic review and meta-analysis is registered on PROSPERO.

This study shall be carried out in line with the Preferred Reporting Items for Systematic Reviews and Meta-Analyses Protocol (PRISMA-P) statement^13^.

### Eligibility criteria

#### Types of studies

All published articles or survey studies with documented or established DRL values in African countries for diagnostic and/or interventional imaging procedures retrievable in the English language will be included in this study. Published articles or survey studies that established the DRL values conducted outside Africa will be excluded.

#### Type of Participants

All individuals exposed to ionizing radiation for medical imaging in Africa.

#### Type of interventions and comparators

There was no intervention, hence the study will not include comparators or a control group.

#### Types of Outcomes

The primary outcome measure is diagnostic reference level (DRLs) in Africa. These will include DRLs specified as, Air kerma-area product (PKA), entrance surface air kerma (Ka.e), kerma at patient entrance reference point (Ka,r), volume computed tomography dose index (CTDIvol), dose length product (DLP) and mean glandular dose (DG) depending on local practices.

### Databases and search strategy

The electronic databases namely; Embase, PubMed, Scopus, Google Scholar, CINAHL, AJOL and Web of Science will be searched using a search strategy developed from 1996 to 2022. Only published articles and survey studies retrieved in English will be included. The search strategy includes MeSH terms, text words, and entry terms. A sample of the PubMed search strategy is displayed in Table 1.

**Table 1:** PubMed search strategy

Recent queries in pmc		
Search	Query	Items found
**#13**	Search ((((“Diagnostic Reference Levels”) OR “Diagnostic Reference Level”)) AND ((x-ray) OR radiography)) AND (((africans) OR african) OR Africa)	61
**#12**	Search (“Diagnostic Reference Levels”) OR “Diagnostic Reference Level”	750
**#11**	Search ((africans) OR african) OR Africa	896855
**#10**	Search africans	34719
**#9**	Search African	633821
**#8**	Search Africa	478200
**#7**	Search (xray) OR radiography	1408405
**#6**	Search radiographs	133781
**#5**	Search radiograph	109229
**#4**	Search radiography	775317
**#3**	Search x ray	1360001
**#2**	Search “Diagnostic Reference Level”	269
**#1**	Search “Diagnostic Reference Levels”	660

### Data collection

#### Study selection

Published articles or survey studies on DRL will be identified through search strategies developed in the databases. The titles and abstracts of potential articles or survey studies will be reviewed and full manuscripts of eligible publications retrievable in English reviewed. The reference lists of related publications will be assessed for possible additional data. Two authors will independently review all selected articles.

Discrepancies will be addressed by a third author as an adjudicator. Review articles, editorial notes or articles published before 1996 will be excluded.

### Data Extraction and Management

Data from articles will be extracted by two authors and compared. Discrepancies will be resolved by a third author as adjudicator. The information that will be extracted includes World Bank income level, World Bank geographical region, country of origin, sponsor, year of publication, age group of the patients, imaging modality, local/regional/national DRL, type of examination, study design, type of DRL quantity, and whether DRLs were established as the 75th percentile or mean of the median or mean DRL quantity measured.

### Statistical Analysis

#### Data synthesis and processing

Descriptive statistics will be formulated and a narrative synthesis of the information of selected studies based on the classification of World Bank income level of the countries, classification of World Bank geographical region, country of origin, sponsor, year of publication, age demography of the patients, imaging modality, local/regional/national DRL, type of examination, study design, type of DRL quantity, and whether DRLs were established as the 75^th^ percentile or mean of the median or mean DRL quantity measured.

### Subgroup analysis

If there is significant heterogeneity, we will conduct a subgroup analysis based on the income level of the countries, geographical regions, and year of publication of DRL.

### Sensitivity analysis

Articles of low quality will be subjected to sensitivity analysis to exclude the effect of low-quality articles.

### Quality of Evidence

The quality of each of the included articles will be assessed independently by two authors using the Effective Public Health Practice Project (EPHPP) tool for quantitative studies. Each article will be rated as low, moderate, or high using the quality assessment scale provided in the EPHPP tool. Discrepancies will be resolved by a discussion between the two authors.

### Risk of Bias

The risk of bias will be assessed using the EPHPP checklist based on the methodological information of each article. We shall evaluate the selection of DRL parameters, method of measurement, reporting of the results and conflicts of interest.

The quality assessment tool score, independently assessed by a pair of reviewers and discrepancies resolved by consensus, will not influence the eligibility of studies. It is merely intended to indicate the quality of the existing evidence base.

## Ethics and Dissemination

This study will not require ethical approval as all data needed for the conduct of the review will be obtained from studies already published in peer-reviewed medical journals. The findings from this study will be published in a high-impact, peer-reviewed journal.

## Discussion

In the last decade, there has been a significant improvement in the quality and safety of medical imaging in Africa through improved awareness of medical imaging safety, application of evidence-based radiation safety recommendations and guideline tools, the establishment of DRL values, and training of end users^3^. However, the radiation safety culture in Africa is still emerging and plagued with several challenges which include limited human resources, inadequate awareness, and low opportunities for education and training^2^,^3^,^11^.

Despite the wide acceptance and recommendation of the DRL process as an essential component for ensuring medical ionizing radiation exposure optimization, there is a relative paucity of data on DRLs in Africa with a population of about 1.216 billion^2^,^11^,^12^. While there is evidence of increased interest in quality and safety in clinical imaging in the last decade, Africa is still faced with major challenges like limited human resources, poor prioritization of imaging in relation to other health services, poor level of awareness for radiation safety, inadequate radiation safety culture, insufficient facilities and opportunities for education and training as a result of a low per capita healthcare budget^2^,^3^,^11^.

This review aims to provide information on the current DRLs status, and the peculiarities surrounding its implementation or non-implementation, raise awareness on the usage of medical ionizing radiation optimization, aid healthcare policies and further research in radiation optimization in Africa.

## Strengths and Limitations of the Study

This will be a comprehensive and exhaustive review of diagnostic reference-level data published in Africa which is a likely strength to this work. The protocol will help establish a greater degree of confidence in the review as it provides step-by-step details in every stage of the systematic review methodology. Furthermore, this study protocol is registered in PROSPERO to reduce redundancy and avoid duplication.

However, similar to other systematic reviews, this study may be limited by the quality of the original individual studies that will be included. Nevertheless, established quality assessment tools to include important methodological criteria that are vital to the validity and interpretation of survey studies will be used to identify relevant original studies. The inclusion process will be made as comprehensive as possible. Another potential susceptibility of this study will be publication bias. However, to reduce the possibility of publication bias in this study, a wide-ranging search for all relevant articles which documented DRLs in Africa will be identified through search strategies developed in five databases with high index of journals in radiology, radiography and medical physics^14^. Furthermore, to alleviate the risk of missing relevant articles, broad headings will be included in the search for each database. The reference lists of related publications will be assessed for possible additional data.

Selection of the articles will be carried out independently by two authors and study authors will be contacted where there is indistinct information.
